# Drying-off practices and cell count–based new infection and cure risk over the dry period on 765 German dairy farms

**DOI:** 10.3168/jdsc.2025-0782

**Published:** 2025-10-18

**Authors:** Andreas R. Böker, Roswitha Merle, Phuong Do Duc, Antonia Hentzsch, Annegret Stock, Frederike Reichmann, Alexander Bartel, Svenja Woudstra, Martina Hoedemaker

**Affiliations:** 1 Clinic for Cattle, University of Veterinary Medicine Hannover, Foundation, 30173 Hannover, Germany; 2 Institute for Veterinary Epidemiology and Biostatistics, Center for Veterinary Public Health, Freie Universität Berlin, 14163 Berlin, Germany; 3 WG Internal Medicine and Surgery, Division for Ruminants and Camelids, Farm Animal Clinic, School of Veterinary Medicine, Freie Universität Berlin, 14163 Berlin, Germany; 4 Clinic for Ruminants with Ambulatory and Herd Health Services, Centre for Clinical Veterinary Medicine, 85764 Oberschleissheim, Germany; 5 Section for Production, Nutrition and Health, Department of Veterinary and Animal Science, University of Copenhagen, 1870 Frederiksberg C, Denmark

## Abstract

This observational study with voluntary participation investigated drying-off practices and dry period-related udder health key performance indicators in 3 important dairy production regions in Germany (the north [NR], east [ER], and south [SR]). Data from DHI testing and information about drying-off practices were collected during a single herd visit between 2016 and 2019 from 253, 252, and 260 farms in NR, ER, and SR, respectively. Abrupt cessation of lactation was most common in NR and ER, but only practiced on about half of the farms in SR (NR: 79%, ER: 75%, SR: 56%). Blanket dry cow therapy (BDCT, the use of antibiotic agents for all cows) dominated in NR and ER (63% and 65%, respectively), whereas in SR, primarily selective dry cow therapy (SDCT, targeted use of antibiotic agents at drying-off for individual cows according to farm-specific criteria; 56%) or even no use of antibiotic dry cow therapy (20%) were adopted. The use of teat sealants (TSL) with or without the use of antibiotics was most common in ER (66%), followed by farms in NR (54%), and least common in SR (30%). The median new infection risk (using a SCC threshold of 100,000 cells/mL of milk) during the dry period (NIRD; cows with SCC ≤100,000 before dry period and SCC >100,000 at first test-day post dry period) in NR, ER, and SR was 24% (interquartile range: 13%–35%), 25% (18%–35%), and 24% (8%–37%), and the cure risk during the dry period (CRD; cows with SCC >100,000 before dry period and SCC ≤100,000 at first test-day post dry period) in NR, ER, and SR was 63% (51%–72%), 57% (47%–65%), and 60% (42%–75%), respectively. The NIRD and CRD varied greatly between farms, indicating improvement potential on many dairy farms.

## Highlights

• Considerable differences in dry cow management were observed between regions.
• Blanket dry cow treatment was still standard in the majority of farms.
• Dry period udder health indicators vary largely between farms.

## Graphical Abstract

**Figure:**
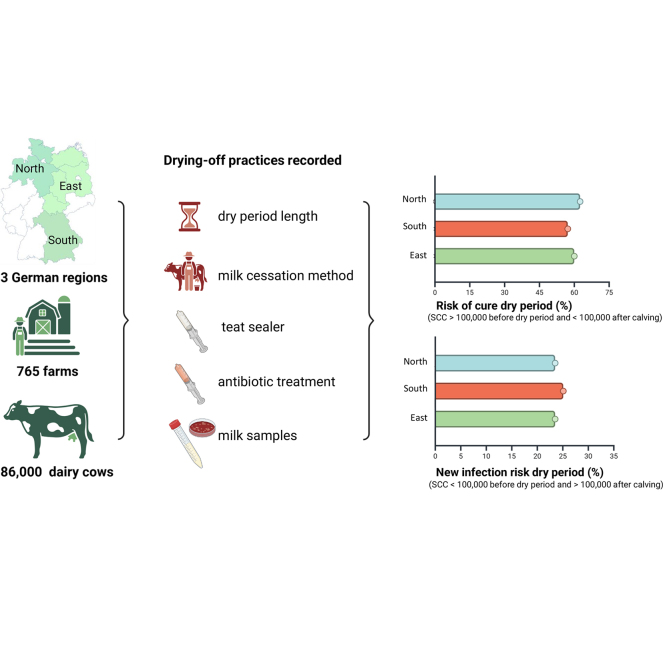
**Summary:** The aim of this study was to investigate drying-off practices and dry period-related udder health key performance indicators in 3 important dairy production regions in Germany (north, east, and south). The duration of the dry period varied considerably between the farms, but had an expected duration of 6 weeks, most common in the north and east, and 8 weeks, most common in the south. Teat sealants were rarely used, and a large number of farms still used blanket dry cow therapy (49.9%). Milk samples taken before drying off were rarely analyzed bacteriologically. The new infection rate in the dry period, with 23.5% to 25.1%, and the cure rate in the dry period, with 57.2% to 62.5%, were similar in the 3 regions. In addition, these rates were comparable to the results of other national and international studies. SCC = somatic cell count. Created in BioRender.

Mastitis is still one of the most common diseases in dairy cows (Ruegg, 2017). During the dry period, many important regenerative processes for the following lactation take place in the udder tissue (Tho Seeth et al., 2015). The dry period offers an ideal opportunity to improve udder health through targeted use of antibiotics for cows with IMI (Halasa et al., 2010). However, prophylactic use of antibiotics for all cows within a herd at drying-off (**BDCT**), originally recommended in the 5-point control plan (Ruegg, 2017), is nowadays discouraged. The Pan-European agreement on dry cow therapy (Bradley et al., 2018) and European Union Regulation 2019/6 (European Parliament, 2018) clearly state that the use of antibiotic dry cow therapy should be minimized where possible. Apart from antibiotic preparations, the use of teat sealant (**TSL**) is recommended. Teat sealant, in addition to the naturally formed keratin plug, closes the teat canal until the start of the following lactation, thus preventing the entry of pathogens and the consecutive new infection of udder tissue (Huxley et al., 2002). The outcome of dry period udder health management can be assessed through key performance indicators (**KPI**) like new infection risk during the dry period (**NIRD**) and cure risk during the dry period (**CRD**), which are both calculated based on changes of the SCC before and after calving (DLQ, 2014).

The aim of this study was to provide a status quo of the udder health situation on German dairy farms by measuring KPI (NIRD and CRD) and collecting detailed data on defined dry-off management practices. Until now, such data have only been reported on a small scale for individual German regions (Bertulat et al., 2015; Sorge et al., 2023).

From December 2016 to July 2019, housing conditions, biosecurity measures, and health situation of German dairy cows were investigated in a large-scale cross-sectional study. In each of 3 German study regions, a sample size of 250 dairy farms was targeted (further information about study design and sampling procedure can be found in Böker et al., 2023). Trained veterinarians visited every farm once and participation in the study was voluntary. In a structured face-to-face questionnaire-based interview, farmers or herd managers (hereafter referred to as “farmers”) were asked about their drying-off practices. Key questions related to the average duration of the dry period, milk cessation method, microbiological examination of milk samples before drying-off, and the use of teat sealants and intramammary antibiotics at the herd level.

Dry period–associated SCC-based udder health KPI were calculated for farms participating in DHI testing (n = 723) in accordance with the German Association for Performance and Quality Testing (DLQ) Guideline 1.15 (DLQ, 2014). The DHI data included all test-day data recorded in the 369 d before the farm visit (i.e., with an average of 11 test dates). The DHI testing was conducted by the German testing organizations. The test data after calving were, therefore, collected for every cow between d 5 to 37 postpartum. Equally, the maximum timespan from last test date to dry-off was 37 d. For this study, information on the individual animal test-day SCC, calving dates, and parity was used. The NIRD was defined as the proportion of animals with an SCC of >100,000 cells/mL of milk on their first test day after calving of all animals with an SCC of ≤100,000 cells/mL of milk at their last test day before drying-off. The CRD was defined as the proportion of animals with an SCC of ≤100,000 cells/mL of milk at the first test day after calving of all animals with an SCC of >100,000 cells/mL of milk at the last test day before drying-off. To enable a comparison of SCC-based udder health indicators in the present study to those observed in other studies, where an SCC limit of 200,000/mL is used as a cut-off for subclinical mastitis, NIRD and CRD were also calculated with a cut-off of 200,000 cells/mL.

All collected data were stored in a Structured Query Language database. Data management and descriptive statistical analyses were conducted in R (version 4.1.2; R Foundation for Statistical Computing). Due to the farms' structural differences between the 3 study regions, a separate analysis was carried out for each region.

A total of 253 farms were visited in the north (**NR**), 252 in the east (**ER**), and 260 in the south (**SR**). The mean number of lactating cows per farm was 105 in NR, 355 in ER, and 45 in SR, with an average milk yield of 9,062 kg/cow per year in NR, 9,222 kg/cow per year in ER, and 7,606 kg/cow per year in SR. A total of 77% (n = 199) of the farms in SR kept more than 80% Simmental cows. In contrast, the Holstein Friesian breed dominated in the other 2 regions (NR: 84% [n = 204]; ER: 79% [n = 198]).

In NR and ER, the most common length of the dry period was 6 wk (35%, n = 89; 36%, n = 90), whereas in SR, it was 8 wk (35%, n = 90, Table 1). Drying-off abruptly was the most common method in NR (79%, n = 199), ER (85%, n = 214), and SR (56%, n = 146). A small number of farms reported an individual cow-dependent milk cessation method (i.e., gradually or abruptly; 6%, n = 15 in NR; 2%, n = 5 in ER, and 6%, n = 16 in SR; Table 1).

**Table 1:** Dry cow management of 765 German dairy farms in 3 different regions

Item	Level	Region		
		North, n (%)	East, n (%)	South, n (%)
Study population		253 (100.00)	252 (100.00)	260 (100.00)
Length of dry period (wk)	<6	22 (8.7)	11 (4.4)	12 (4.6)
	6	89 (35.2)	90 (35.7)	67 (25.8)
	7	81 (32.0)	49 (19.4)	84 (32.3)
	8	53 (21.0)	82 (32.5)	90 (34.6)
	>8	7 (2.8)	19 (7.5)	7 (2.7)
	No answer	1 (0.4)	1 (0.4)	0 (0.0)
Cessation type^1^	Abrupt	199 (78.7)	214 (84.9)	146 (56.2)
	Gradual	39 (15.4)	33 (13.1)	98 (37.7)
	Mixture	15 (5.9)	5 (2.0)	16 (6.2)
Use of antibiotic dry cow therapy	All cows	158 (62.5)	163 (64.7)	61 (23.6)
	Selective	81 (32.0)	69 (27.4)	146 (56.2)
	Not used	14 (5.5)	20 (7.9)	52 (20.0)
	No answer	0 (0.0)	0 (0.0)	1 (0.4)
Use of teat sealers at drying-off	All cows	95 (37.6)	143 (56.8)	35 (13.5)
	Selective	41 (16.2)	24 (9.3)	44 (16.9)
	Not used	117 (46.3)	85 (33.7)	180 (69.2)
	No answer	0 (0.0)	0 (0.0)	1 (0.4)
Bacteriology of milk samples before drying-off	>80% of cows	6 (2.4)	25 (9.9)	17 (6.5)
	50%–80%	3 (1.2)	9 (3.6)	17 (6.5)
	<50% of cows	55 (21.7)	43 (17.1)	38 (14.6)
	Never	185 (73.1)	175 (69.4)	187 (71.9)
	No answer	4 (1.6)	0 (0.0)	1 (0.4)

1 Gradual: slowly reduce milking frequency (for all cows at dry-off); mixture: individual for every cow (gradual or abrupt cessation); abrupt: sudden ending of lactation by not milking anymore.

Antibiotic therapy at drying-off was frequently used on the farms visited. In NR, 63% (n = 158) of the farms used BDCT (the use of antibiotic agents at drying-off for all cows). This proportion was slightly higher in ER (65%; n = 163), whereas it was lower in SR (24%; n = 61). In SR, selective dry cow therapy (**SDCT**, targeted use of antibiotic agents at drying-off for individual cows according to farm-specific criteria) dominated with 56% (n = 146), whereas 20% (n = 52) of the farmers stated that they did not use any antibiotic agents for drying-off. Only 14 (6%) farms in NR and 20 (8%) farms in ER dried off all cows without using antibiotics (Table 1).

General use of TSL for all cows at drying-off was most common in ER (57%, n = 143). In contrast, about one-third (n = 85) of the farms in ER did not apply TSL at all, whereas 9% (n = 24) used TSL selectively. Application of TSL for all cows was practiced in 38% (n = 95) of all farms in NR and almost half (n = 117; 46%) did not use any TSL for drying-off. Only 13% (n = 35) of the farmers in SR dried off all their animals with TSL (Table 1); 69% (n = 180) did not use TSL for drying-off. As shown in Figure 1, there are regional differences in the combined use of antibiotics and TSL. Both an antibiotic agent and a TSL were applied by 21% (n = 54) of farmers in NR, 40% (n = 100) in ER, and only 4% (n = 10) in SR for drying-off all their cows. The most dominant drying-off practice in NR was BDCT without use of TSL (36%, n = 92) and SDCT without the use of TSL in SR (34%, n = 88).

**Figure 1:**
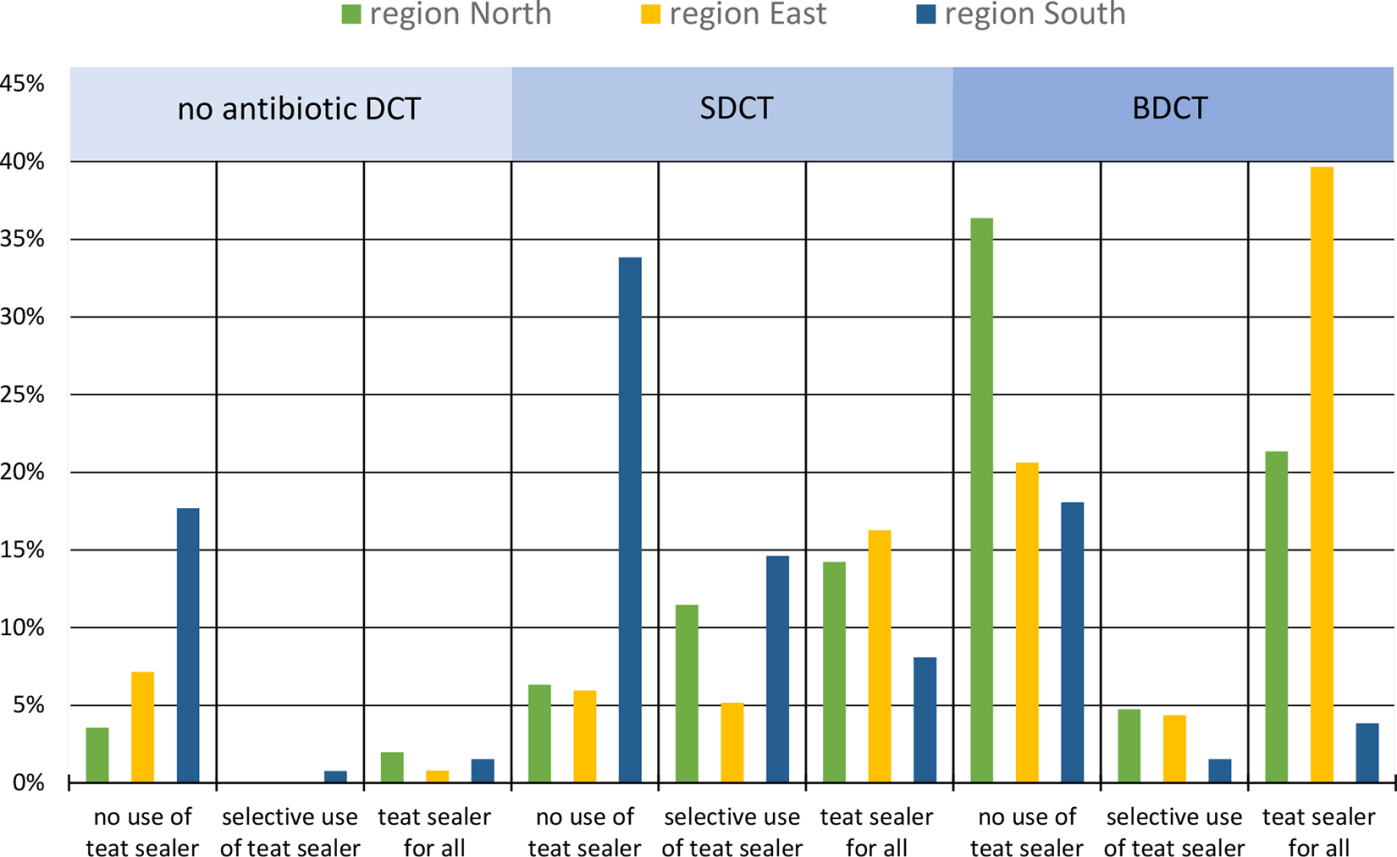
Percentage of German farms using different combinations of antibiotics and teat sealer for dry cow therapy sorted by region. No antibiotic DCT = dry cow therapy; SDCT = selective dry cow therapy; BDCT = blanket dry cow therapy (the use of antibiotic agents at drying-off for all cows).

Microbiological examination of milk samples was not routinely performed before drying-off in any region. Around 13% of the farms in ER (n = 34) and SR (n = 34) and only 4% (n = 9) in NR collected milk samples from more than 50% of their cows before drying-off. About 70% of the farms in all 3 regions never took a milk sample of any cow for microbiological examination before drying-off (Table 1).

The median NIRD at herd level with an SCC cut-off of 100,000 cells/mL of milk was 24% (interquartile range [**IQR**]: 13%–35%) in NR, 25% (IQR 18%–35%) in ER, and 24% (IQR 8%–37%) in SR (Table 2). The median CRD with an SCC cut-off of 100,000 cells/mL of milk at herd level was 63% (IQR 51%–72%) in NR, 57% (IQR 47%–65%) in ER, and 60% (42%–75%) in SR (Table 2). The NIRD with BDCT was noticeably lower compared with farms where antibiotics are not used at all (NR: −8%, ER: −13%, SR: −15%). The CRD was much higher with BDCT than when no antibiotics were used (NR: +19%, ER: +18%, SR: +25%). The values for SDCT lie between these 2 extremes, with CRD closer to the BDCT result and NIRD closer to the values without antibiotic use. However, when looking at the average NIRD and CRD, it is noticeable that they are almost similar in the 3 regions.

**Table 2:** Cure risk (CRD) and new infection risk (NIRD) during the dry period

SCC limit value	Item	Region	n^1^	Q 0.1^2^ (%)	Q 0.25^2^ (%)	Median^3^ (%)	Q 0.75^2^ (%)	Q 0.9^2^ (%)	Mean^4^ (%)
100,000	CRD	North	242	42.3	51.1	62.5	72.2	85.2	62.4
		East	249	34.8	46.6	57.2	64.9	73.4	54.9
		South	232	23.3	41.8	60.0	75.0	87.5	58.3
200,000	CRD	North	239	54.2	65.0	75.2	83.3	96.3	75.1
		East	248	45.4	57.1	67.6	76.7	83.9	66.3
		South	232	33.3	50.0	71.4	87.5	100.0	69.2
100,000	NIRD	North	242	0.0	12.7	23.5	34.5	46.4	24.5
		East	249	13.1	18.0	25.1	35.4	50.2	29.2
		South	232	0.0	7.8	23.5	37.4	50.0	24.5
200,000	NIRD	North	239	3.1	8.9	14.6	22.9	30.4	16.7
		East	248	8.3	12.7	17.0	23.5	33.0	19.1
		South	232	0.0	5.6	14.3	24.6	33.3	16.4

1 Number of farms participating in DHI testing.

2 Q 0.1, 0.25, 0.75, and 0.9 = average values of test-day quantiles 10%, 25%, 75%, and 90%, respectively.

3 Average values of annual test-day median (Q 0.5) at the farm level.

4 Average values of annual test-day mean at the farm level.

This is the first cross-sectional study investigating the prevalence of SCC-based indicators of dry cow udder health in combination with drying-off practices in a representative sample of several major dairy regions in Germany. The aim of the study was to provide benchmarks for farmers and their advising veterinarians and to document the status quo of drying-off management.

A sufficient period for recovery of the mammary gland tissue is essential for udder health in the following lactation. A dry period of more than 30 d correlates with higher milk yield in the following lactation (Niemi et al., 2022). The recommended duration of the dry period is 45 to 60 d (Tho Seeth et al., 2015). Within our study population, the length of the dry period varied greatly. Of 765 farms, 57% (n = 439) followed the recommended length. In contrast, 38% (n = 291) favored a shorter period of 6 wk or less, with the risk of insufficient time for optimal udder tissue regeneration. Only 4% (n = 33) of the farms chose a dry period of more than 8 wk. Our results, which show considerable variation in dry period length, are similar to those of the studies by Bach et al. (2022) and Niemi et al. (2022). Others had less variability in the dry period length and even more farms were in the range of 45 to 60 d (Bertulat et al., 2015; Vilar et al., 2018). Significantly longer times have also been documented (Clabby et al., 2022).

On assessing the milk cessation method, our data showed that almost three-quarters of the farms dried off their cows abruptly, whereas only less than a quarter dried them off gradually. However, this proportion of gradual drying-off was mainly found on farms in SR, which are on average much smaller, have a lower milk yield, use more frequently tethered housing, and keep a different breed (mainly Simmental) than the farms in NR and ER. The high proportion of farms with abrupt drying-off in our results was almost identical to Bertulat et al. (2015) on northern German dairy farms. In Switzerland, where farms are also usually smaller, Bach et al. (2022) found a proportion of abrupt drying-off of 45%, similar to our observation for SR. Gradual drying-off is a practical way of significantly reducing milk yield before cessation of lactation (Bach et al., 2022; Rajala-Schultz et al., 2018). It accelerates the involution of the mammary glands and increases the natural protective factors of the udder (including the rapid formation of a keratin plug). Milk yields of 15 kg/d or less significantly reduce the risk of new IMI (Rajala-Schultz et al., 2018). In addition, animal welfare is less impaired when a cow is gradually dried off at a lower yield (Bach et al., 2022).

Use of BDCT was widespread among the farms surveyed, although noticeably less used in SR than in ER and NR. Overall, BDCT was reported as standard practice by 50% of all farms. This value is lower than that of 80% reported by Bertulat et al. (2015) for northern Germany. A French study also reported a slightly higher value of 58% (Poizat et al., 2017). In North America, values between 78% and 94% (USDA, 2016; Bauman et al., 2018) have been reported. The Finnish study by Vilar et al. (2018) had by far the lowest prevalence of BDCT of only 13%. These differences are probably due to different perspectives regarding antibiotic use for BDCT, national regulations limiting the use of antibiotics, and trends over time affecting the use of antibiotics and their acceptance in society.

Since 2022, the prophylactic use of antibiotics has been prohibited by European Union Regulation 2019/6 (European Parliament, 2018). It is therefore the responsibility of veterinarians and farmers to minimize the use of antibiotics in an evidence-based manner. McCubbin et al. (2023) found, that with SDCT, antibiotic consumption was reduced by about 50%. There are no negative effects on udder health when SDCT is practiced correctly (Cameron et al., 2015). Even greater reductions in antibiotic consumption are therefore conceivable. In the study population, SDCT was implemented on 39% of the study farms, and 11% of farmers stated that they did not use antibiotics for drying-off at all. In other studies, SDCT was used less often, for example, 31% in Canada (McCubbin et al., 2023), whereas there were also studies in which 52% (e.g., Switzerland), 74% (e.g., Finland), or 78% (e.g., Austria) of the farms already used SDCT (Bach et al., 2022; Vilar et al., 2018; Firth et al., 2019, respectively). In a French study from 2017, comparable results with our findings (42%) were described for the implementation of SDCT (Poizat et al., 2017). Our study was conducted before the EU Regulation 2019/6 came into force and therefore gives an estimate for the level of implementation of SDCT in Germany before 2022.

Antibiotic dry cow therapy is used to treat IMI at dry-off and dry cow treatment is justified when the cure rate after antibiotic treatment exceeds the expected rate of spontaneous cure. Müller et al. (2023) recently found that only infections with a few pathogen species, particularly *Streptococcus* spp., benefit from using antibiotic dry cow treatment. In contrast, infections with NAS have rather short infection durations (Woudstra et al., 2023) and therefore likely do not benefit from antibiotic treatment during the dry period. Therefore, SDCT of only cows with treatment-worthy infections is recommended. Microbiological examination of milk, in addition to SCC measurements, can be very useful in selecting cows or quarters benefiting from antibiotic treatment, thus avoiding unnecessary treatment (Rowe et al., 2023). However, 72% of all farms in this study (NR: 73%, ER: 69%, SR: 72%) never took samples for detecting IMI before drying-off, whereas in Austria, Firth et al. (2019) only reported a rate of 18% of farms never performing bacterial culture before drying-off. Nonetheless, as on-farm sampling and culture are costly, preselecting quarters for microbiological testing by using a California Mastitis Test or DHI test results is a reasonable method (Sargeant et al., 2001).

Intramammary TSL can be used alone or in combination with intramammary antibiotic treatment at drying-off. A total of 36% of the farms used TSL for all of their cows. McCubbin et al. (2023) reported that 56% of Canadian dairy farms applied TSL to all cows and a further 12% did so selectively. Several studies have focused on the efficacy and benefits of TSL in dry cow treatment and most of them confirmed good efficacy in terms of preventing new infections during the dry period, especially when TSL is used alone without antibiotics (McCubbin et al., 2022). Some studies have observed a slight increase in SCC in the following lactation when TSL was used without antibiotics (McParland et al., 2019; Clabby et al., 2022). However, Huxley et al. (2002) found that drying-off with the application of a TSL alone even resulted in lower NIRD by major pathogens, and McParland et al. (2019) even documented a slight increase in milk yield in the following lactation when using TSL alone.

The combination of antibiotic dry cow therapy and TSL is very effective in protecting against new cases of IMI until calving (Godden et al., 2003). However, it is also partly dependent on the bacteria causing new IMI as to whether TSL supports antibiotic dry period treatment or not (Afifi et al., 2023). Nonetheless, we showed that the combination of targeted antibiotic SDCT and general use of TSL was rarely implemented.

The median NIRD of 24% to 25% determined for the different regions in this study was very similar to the median NIRD of 23% found by Sorge et al. (2023). In the present study, the NIRD was 17 to 30 percentage points higher in the 25% weakest compared with the 25% best farms, which indicates a large potential for udder health improvements during the dry period and early lactation in many dairy cow herds. Similar variations have also been reported previously for individual German regions (Sorge et al., 2023).

The median CRD of 57% to 63% was slightly lower than the CRD of 64% reported by Sorge et al. (2023). The difference in CRD between quantile 0.25 and quantile 0.75 ranged from 18 to 33 percentage points, depending on the region. These findings also underline the potential of improvement in CRD. It should be noted that the CRD not only refers to the healing of existing infections but is also influenced by re-infection of initially healed udders during the dry period. A distinction between ongoing and new infections, however, is not possible based on SCC measurements only, but could only be made through bacteriological culture in combination with strain typing, which is currently not applicable in practice.

In summary, there were considerable regional differences in on-farm dry cow management. A large proportion still used BDCT. Teat sealants were commonly used in ER, but very little in SR. Microbiological testing of milk samples before drying-off was insufficiently used regarding the potential of evaluating the need for antibiotic treatment. Considering the present overall aim of limiting antibiotic consumption, a switch to SDCT is crucial and necessary. The data collected in this study show a status quo that can also be used by German dairy farmers and their veterinarians as a benchmark for assessing the udder health situation in their herds. The observed KPI (NIRD, CRD) varied considerably between individual farms within geographical regions (between the worst and the best), although there were no major differences between regions, indicating a high potential for further improvements in udder health in dairy cows in many German herds.
